# Application value of plasma Neurofilament light combined with magnetic resonance imaging to comprehensively evaluate multiple sclerosis activity and status

**DOI:** 10.3389/fneur.2023.1295904

**Published:** 2023-11-24

**Authors:** Feiyue Mi, Yingchun Wang, Wenqiang Chai, Ya Chen, Xuhua Yin

**Affiliations:** ^1^Department of Neurology, Affiliated Hospital of Inner Mongolia Medical University, Hohhot, Inner Mongolia Autonomous Region, China; ^2^General Hospital of Inner Mongolia Autonomous Region of the Chinese People's Armed Police Force, Hohhot, Inner Mongolia Autonomous Region, China; ^3^Department of Neurology, Affiliated Hospital of Zunyi Medical University, Zunyi, Guizhou province, China

**Keywords:** multiple sclerosis, Neurofilament light, magnetic resonance imaging, activity, biomarkers

## Abstract

**Objective:**

Compare the levels of plasma neurofilament light (NfL) in patients with multiple sclerosis (MS) at acute and remission stages and healthy individuals to explore the role of plasma NfL in monitoring the activity and severity of the disease and predicting disease prognosis.

**Methods:**

Information on healthy individuals and patients with MS who visited the outpatient and inpatient departments of Inner Mongolia Medical University Affiliated Hospital from October 2020 to August 2022 was collected. EDSS assessment and plain scan+enhanced magnetic resonance imaging (MRI). Plasma Nfl levels were measured using Simoa. Moreover, the relationship between the level of Nlf and the disease status of patients with MS was analyzed..

**Results:**

Through the self-comparison of the plasma NfL levels of MS patients in the acute and remission stages, it was noted that the levels in the acute stage are higher than those in the remission stage (*p* < 0.001). Among the plasma NfL levels of healthy individuals and MS patients in the acute and remission stages, there were statistically significant differences (*p* < 0.001). Furthermore, the plasma NfL level did not correlate with age or course of disease (*p* = 0.614 and *p* = 0.058), whereas it correlated with EDSS score, the number of MRI T2 subtentorial and spinal cord lesions, and the number of MRI enhanced lesions (*r* = 0.789, *p* < 0.001; r = 0.846, *p* < 0.001; *r* = 0431, *p =* 0.005, respectively).

**Conclusion:**

Combining the level of plasma NfL with clinical and MRI estimations will be instrumental in monitoring condition changes and optimizing treatments. The level of plasma NfL is related to the activity and severity of MS, and it is expected to become a new biomarker for assessing the activity and disease status of MS.

## 1 Introduction

Multiple sclerosis (MS) is a chronic degenerative autoimmune disease of the central nervous system (CNS), with inflammation, demyelination, and axis cylinder loss occurring in the early stage (1). Differential involvements of motor, sensory, visual, and autonomic nervous systems can cause a series of symptoms and signs, such as limb weakness, sensory abnormalities, impaired vision, and dystaxia (2), which are the main etiologies of disability among young people globally, placing a significant burden on the social economy (3). The Global Burden of Disease (GBD) research team of the Institute of Health Metrics and Evaluation of the University of Washington discovered that there were approximately 2.3 million MS cases globally, with an increase of 10.4% in morbidity since 1990. Between 1990 and 2016, the prevalence rates in Chinese Mainland and Taiwan rose by 45.6 and 49.5%, respectively. Approximately three-quarters of the global MS patients are women (4). In 2018, China marked MS as a rare disease. In 2020, MS incidence rates in China were released for the first time: 0.235/100,000 per year, 0.055/100,000 in children, and 0.288/100,000 in adults. The geographical distribution of MS shows an east–west altitude gradient. Residents in high latitude and altitude areas tend to develop MS. The incidence rate in Inner Mongolia is more than 0.4/100,000, ranking first in China (5).

MS has multiple temporal and spatial clinical features. At present, the 2017 McDonald criteria is recommended, and the diagnosis mainly depends on clinical manifestations, cerebrospinal fluid and MRI (6). The abovementioned indicators are also applied in prognostic evaluation. However, assessing the prognoses of patients with MS only based on clinical and imaging evidence is hysteretic. Therefore, clinicians need new highly specific and sensitive biomarkers that can monitor disease activity and severity and indicate a prognosis to comprehensively understand MS, implement precise and personalized management of patients with feasible methods as much as possible, improve prognosis, raise patients’ quality of life, and lighten the economic burden on patients.

Neurofilament (NF) is a specific component of the cytoskeleton, primarily maintaining the elasticity and integrity of nerve fibers and ensuring their functions. NF includes Neurofilament light (NfL), Neurofilament medium (NfM), Neurofilament heavy (NfH), α-endonuclease, and type-III peripherin. When CNS axons are injured, the NF released into the cerebrospinal fluid indicates axonal injury and neuronal death (7). Under normal physiological conditions, low concentrations of NfL are released from axons into the cerebrospinal fluid and enter the bloodstream through the blood–brain barrier (BBB) at low concentrations. The release of NfL gradually increases with age (8). If affect neuronal axonal damage, the release process will be accelerated, which is the basis for using NfL as a marker for axonal damage (9, 10). Williams et al. (11) found a strong correlation between NfL and disease activity. As an ongoing quantitative measurement of axonal injury, the increase in NfL may be conducive to the prognosis of neurological diseases (12).

When the CNS axonal injury occurs, NfL is first released into the cerebrospinal fluid, and a small portion enters the peripheral blood through the BBB. The content of NfL in the peripheral blood is 40 times lower than that in the cerebrospinal fluid. In the past, NfL detection was mainly based on cerebrospinal fluid measurement using the ELISA method. However, the traditional ELISA method usually fails to detect it. The advanced detection method, Simoa, has a sensitivity 1000 times higher than traditional ELISA, making it possible to plasma NfL. Moreover, NfL sampling in the peripheral blood is easy and convenient to store, allowing for testing (13). It has become a trend of replacing cerebrospinal fluid NfL with plasma NfL.

Relapsing remitting multiple sclerosis (RRMS) is a recurrent process. Nerve fiber damage and axonal loss are the main mechanisms causing neurological disability. Therefore, simple and direct multimode methods are required to monitor disease changes and perform follow-up. NfL may play a specific role in monitoring the activity, severity, treatment efficacy, and prognosis of MS as a marker of axonal injury.

## 2 Experimental materials and methods

### 2.1 Research subjects

This study included 42 patients with MS who visited the outpatient and inpatient departments of Inner Mongolia Medical University Affiliated Hospital from October 2020 to August 2022, including 25 patients in the acute stage and 17 patients in the remission stage. All patients with MS met the 2017 edition of the McDonald’s criteria and were classified as relapsing–remitting. As healthy controls, 26 healthy individuals were included, aged from 20 to 60 years. All enrolled patients signed an informed consent form, and all research procedures were approved by the Ethics Committee (Inner Mongolia Medical University Affiliated Hospital, NO. WZ2023056).

**Figure 1:**
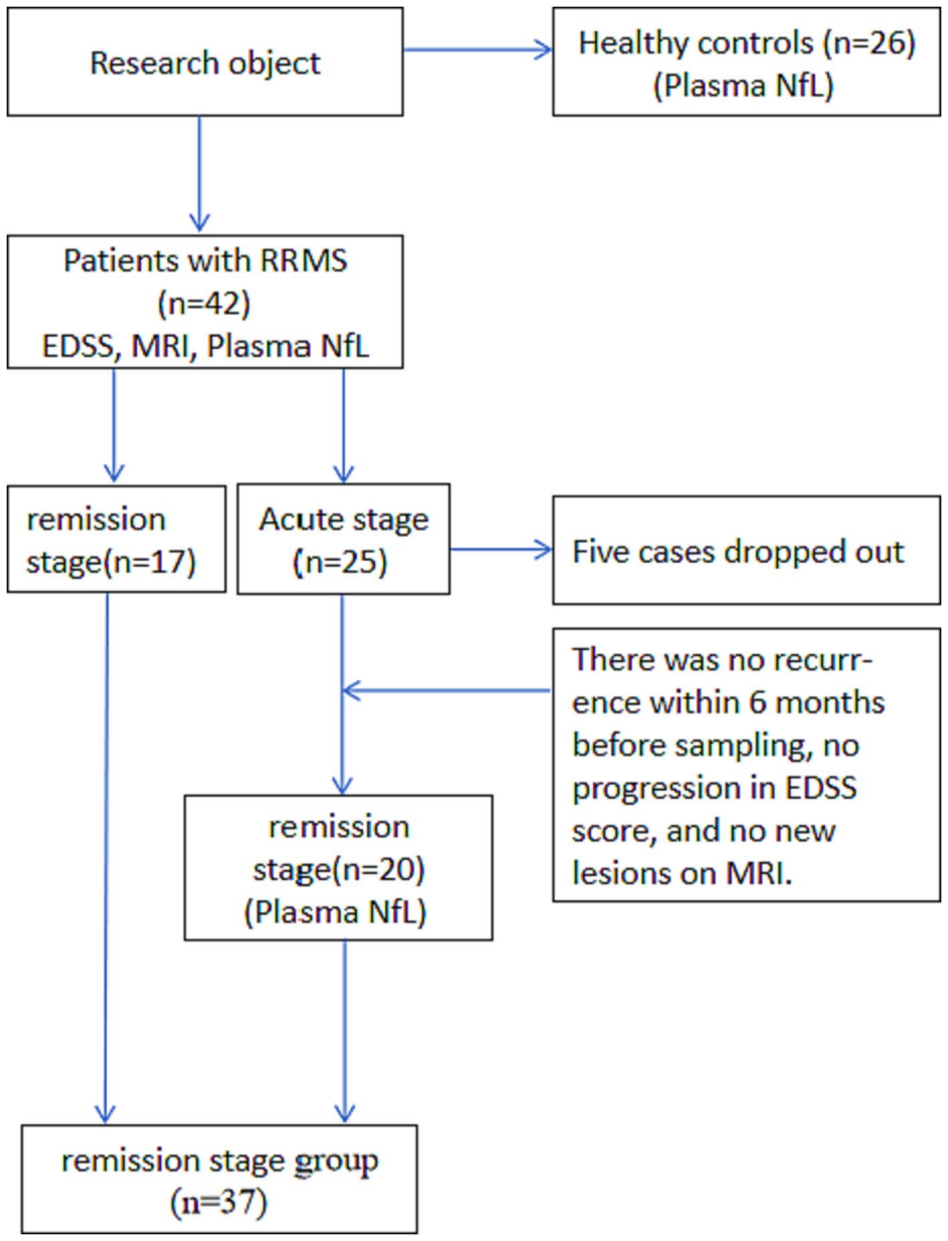
Flowchart.

The enrolled 25 acute patients were treated with steroid pulse or intravenous immunoglobulin therapy and improved. After discharge, 20 patients were followed up within 6 months. The patients without new clinical symptoms or enhanced MRI lesions were classified as in the remission stage and underwent plasma NfL testing. Those who recurred or had enhanced lesions were excluded. For the present study, the acute stage was regarded as the current event with pathological changes in the CNS acute inflammatory demyelination. It was found according to the subjective description of patients or objective examination and lasted more than 24 h and less than 1 month. The remission stage was regarded as no recurrence within 6 months before sampling, no progression in EDSS assessment, and no new lesions found on MRI scans. Five cases dropped out of the study: one who went to study abroad, two who changed their residences, and two who were lost due to the Covid-19 pandemic (Figure 1).

#### 2.1.1 Inclusion criteria

1. The seizure type and clinical symptoms of MS complied with the revised McDonald criteria in 2017. After a detailed inquiry of medical history, physical examination, and head MRI examination, the patient was diagnosed with MS;
2. Aged 18–60 years;
3. No recurrence within 6 months before sampling, no progression in EDSS assessment, and no new lesions on MRI scans;
4. Those who were able to complete survey scales and MRI;
5. The patient and their family agreed to participate in this study and signed an informed consent form.

#### 2.1.2 Exclusion criteria

1. Those with clear liver and kidney function damage, hypertension, diabetes, and heart disease;
2. Combined with fever, elevated hemogram, and other infectious symptoms;
3. Concomitant cerebrovascular disease and tumor;
4. Concomitant peripheral neuropathy, motor neuron disease, and Parkinson’s disease;
5. Concomitant diseases that affect information collection, such as dementia and mental illness, and those who were not interested in participating in this study.

### 2.2 Experimental methods

MS patients were required to complete their basic information, including age, gender, body mass index (BMI), and course of disease, and plasma NfL testing were carried out. Patients who were followed up within 6 months to 1 year also underwent the abovementioned examinations. Healthy individuals improved their basic information and underwent the plasma NfL testing.

#### 2.2.1 EDSS evaluation and imaging examination

**Figure 2:**
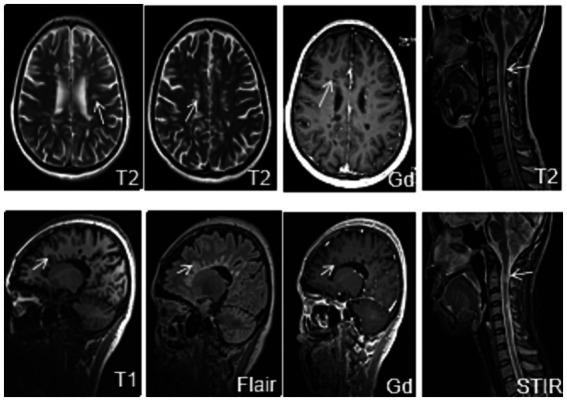
Lesions on MRI.

On the day of blood collection, two professional neurologists conducted EDSS evaluations on all patients with MS, and scores were given. The scoring criteria were strictly followed to decline errors. All MS patients underwent a plain scan and enhanced 3.0 T MRI on the head, cervical, and thoracic spinal cord. With the assistance of professional neurologists and imaging neurologists, the presence and the number of enhanced lesions were checked, as well as the number of T2 subtentorial and spinal cord lesions (Figure 2).

#### 2.2.2 Plasma NfL detection

**Figure 3:**
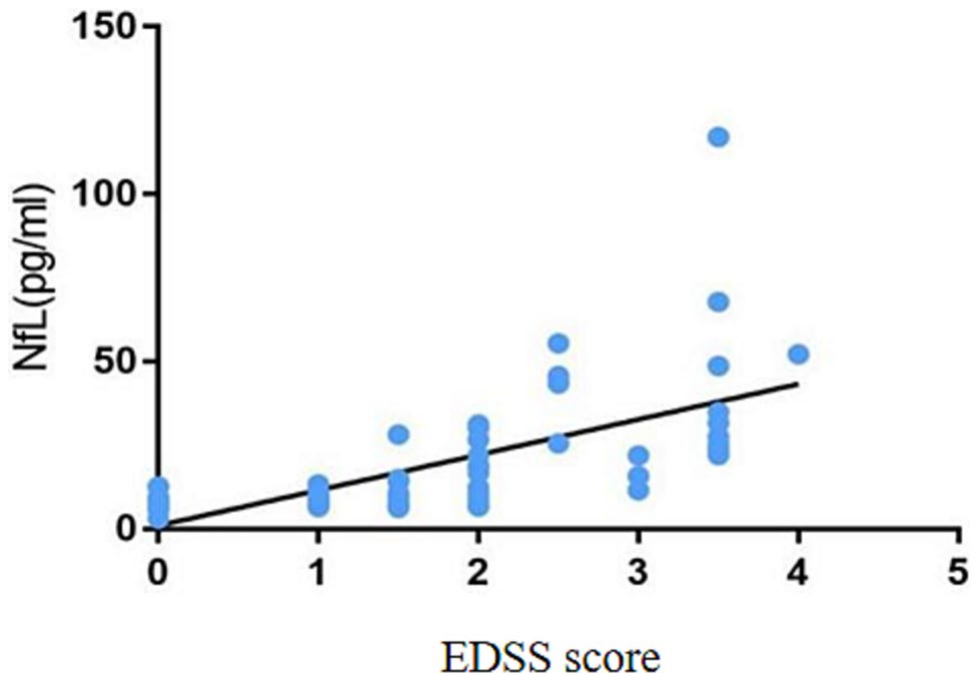
Correlation between EDSS score and the plasma NfL level.

During blood collection, 5 mL of fasting peripheral blood was extracted from all participants using EDTA tubes. The blood was centrifuged at 1000 rpm for 10 min, and then the upper layer was extracted. The plasma sample was transferred to a 96-well plate, shaking magnetic beads for at least 30 s. The machine was turned on to preheat and entered the NfL detection program. The Simoa platform was used for batch or individual testing. Finally, a report of the results was generated and exported in PDF format for subsequent analysis (Figure 3).

### 2.3 Statistical methods

**Figure 4:**
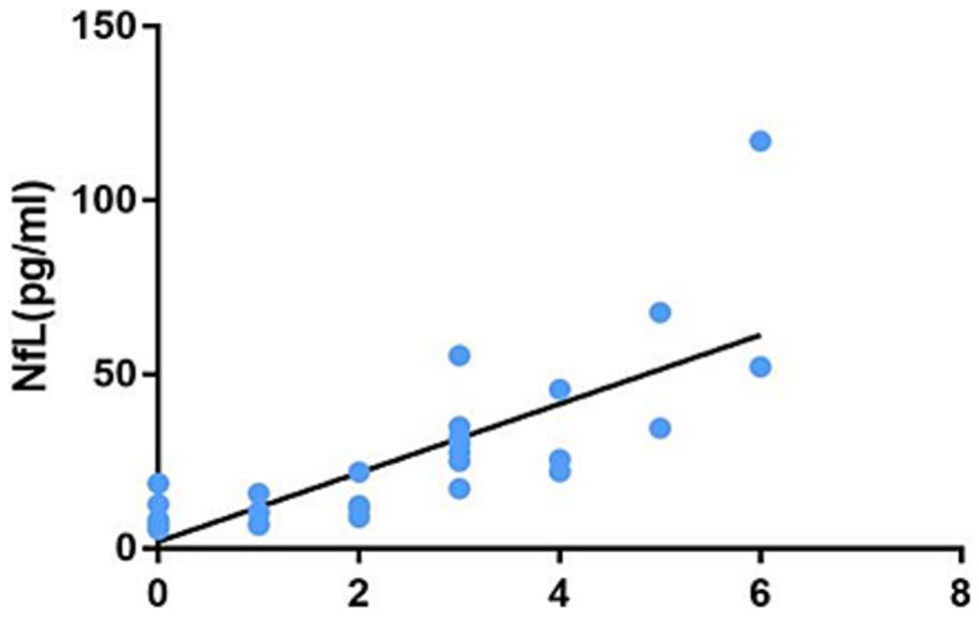
Correlation between the number of infratentorial and spinal cord lesions on MRI and the plasma NfL level.

All data were analyzed using SPSS26.0. Normally distributed measurement data were described as mean ± standard deviation. A T-test was used for two groups, and analysis of variance was used for multiple groups. The measurement data of non-normal distribution were represented by median and interquartile intervals, and non-parametric tests were used: K-W test or Wilcoxon test. The enumeration data were expressed as composition ratio, and the *X*^2^ test was used for comparison between groups. Spearman correlation analysis was used. The significance level was *α* = 0.05, with *p* < 0.05 considered as statistically significant (Figure 4).

## 3 Results

### 3.1 Basic information

This study screened and incorporated 42 patients with RRMS, aged between 20 and 60 years, with an average age of 40 years. As controls, 26 healthy individuals were included. The basic information on patients with MS and healthy individuals is shown in Table 1. There was no statistically significant difference in age, sex, and BMI between the two groups (*p* > 0.05). The results were comparable.

**Table 1:** Basic information of patients with MS and healthy individuals.

	MS	CG	T/χ^2^	*P*
Sample size	42	26		
Age (year, x ± S)	40.12 ± 12.45	40.81 ± 12.92	−0.219	0.828
Sex (male/female)	12/30	7/19	0.022	0.883
BMI (kg/m^2^, x ± S)	23.99 ± 3.68	23.94 ± 2.62	0.058	0.954

Statistical description and analysis were conducted on the course of the disease, EDSS score, number of infratentorial and spinal cord lesions on MRI, and number of enhanced MRI lesions of the two groups. The results are shown in Table 2. Between the two groups, there was no statistically significant difference in the course of disease (*p* = 0.523), whereas the EDSS score and number of enhanced lesions on MRI had statistical significance (*p* < 0.001) and the number of MRI T2 infratentorial and spinal cord lesions also had statistical significance (*p* = 0.037).

**Table 2:** Comparison of clinical data of patients with MS.

Indicator	Acute stage (*n* = 25)	Remission stage (*n* = 37)	*Z*	*P*
Course of disease (month)	46.0 (17.5, 101.5)	57.0 (26.5, 87.5)	−0.639	0.523
EDSS score	2.5 (2.0, 3.5)	1.0 (0, 1.5)	−5.137	<0.001
Number of MRI T2 infratentorial and spinal cord lesions	3.0 (1.0, 4.0)	0.5 (0, 1.8)	−2.081	0.037
Number of MRI-enhanced lesions	1.0 (1.0, 2.0)	0 (0, 0)	−7.142	<0.001

### 3.2 Plasma NfL comparison between MS patients in acute and remission stages and healthy individuals

To further analyze plasma NfL levels of different stages of MS and healthy individuals, the study subjects were divided into three groups for intergroup comparison. The differences in plasma NfL levels were statistically significant (Table 3).

**Table 3:** Comparison of plasma NfL levels between MS acute and remission stages and healthy individuals.

	NfL(pg/ml)	*H*	*P*
Acute stage(*n* = 25)	21.96 (10.8933.12)^a,b^		
Remission stage (*n* = 37)	9.43 (7.4515.81)^c^	23.856	<0.001
Healthy individuals group(*n* = 26)	7.90 (3.83,10.63)		

^a^indicates a significant difference between the acute stage and the remission stage; ^b^denotes a significant difference between the acute stage and the healthy individual group; ^c^represents a significant difference between the remission stage and the healthy individual group.

### 3.3 Plasma NfL comparison between MS patients in acute and remission stages

A self-control study was conducted on the changes in the plasma NfL levels of the acute and remission stages of 20 MS patients who were followed up and re-examined after treatment in the acute stage. The results are shown in Table 4. There was statistical significance in plasma NfL levels between the acute and remission stages of patients with MS (*p* < 0.001).

**Table 4:** Comparison of plasma NfL levels between acute and remission stages in patients with MS.

	NfL(pg/ml)	*Z*	*P*
Acute stage (*n* = 20)	23.54 (13.1734.12)	−3.696	<0.001
Remission stage (*n* = 20)	12.05 (8.6818.33)		

### 3.4 Correlation analysis of NfL in MS patients

This study analyzed the correlations between the plasma NfL level and age, course of disease, EDSS score, number of infratentorial and spinal cord lesions on MRI T2, and MRI-enhanced lesions. The results are shown in Table 5. The plasma NfL level is not correlated with age and course of disease (*p* = 0.614 and *p* = 0.058). However, it had correlations with EDSS score, the number of infratentorial and spinal cord lesions on MRI T2, and the number of MRI enhanced lesions (*r* = 0.789, *p* < 0.001; *r* = 0.846, *p* < 0.001; *r* = 0431, *p* = 0.005, respectively).

**Table 5:** Correlation analysis of NfL in patients with MS.

	NfL level	
Indicator	*r*	*p*
Age	−0.065	0.614
Course of disease	−0.243	0.058
EDSS score	0.789	<0.001
Number of MRI T2 infratentorial and spinal cord lesions	0.846	<0.001
Number of enhanced lesions	0.495	0.005

## 4 Discussion

MS is a complex heterogeneous disease with unpredictable course and prognosis. In the past few decades, the clinical application of biomarkers in neurodegenerative diseases has gradually increased. Scholars have disclosed that plasma NfL is a promising indicator for axonal injury.

Research on NfL began with cerebrospinal fluid specimens. NfL in the cerebrospinal fluid possesses potential prognostic value in clinical isolation syndrome (CIS) and RRMS and can be a disease activity biomarker (14). Gaetani et al. collected cerebrospinal fluid samples from 32 patients with MS within 30 days after the first demyelination event and followed them up for 3.8 ± 2.5 years. The cerebrospinal fluid NfL was measured using ELISA. In the first demyelination event, patients with subsequent disease activities had higher baseline cerebrospinal fluid NfL values than clinically and radiologically stable patients (15). Scholars outside China have recognized a high correlation between blood and the cerebrospinal fluid NfL level, indicating that blood sampling can replace cerebrospinal fluid collection. Repeated measurement of NfL in the peripheral blood to detect axonal damage may become a new approach for monitoring MS (16). This experiment compared the NfL levels of 20 patients with MS. It was found that the NfL levels of patients in the acute stage were significantly higher than those of patients in the remission stage, with significant differences. The plasma NfL levels in both the acute and remission stages of MS were higher than those in healthy individuals, implying a significant increase in NfL during the acute stage of the disease, further demonstrating that NfL, as a marker of neuronal axonal injury, is always associated with inflammatory activity in MS, and has important significance in monitoring disease activity.

In addition to inflammatory activity, another related aspect of MS pathology is the occurrence of neurodegeneration and progressive disability, which can also be objectively measured using NfL. Research has confirmed that patients with progressive MS have higher NfL levels than age and sex-matched recurrent patients (17). Generally, in contrast with RRMS, patients with progressive MS are more severe, with more functional lesions in the neurological system. A study incorporated 41 patients with CIS, 34 patients with MS, 73 patients with neuromyelitis optica spectrum disease as the patients groups; 40 lumbar puncture patients diagnosed with neurosis and migraine as the normal control groups. The clinical and neuroimaging features of the MS group and cerebrospinal fluid samples from the two groups were collected. An enzyme-linked immunosorbent assay was used to measure the NfL level in the cerebrospinal fluid of patients in each group. The NfL levels in the CSF of CIS, MS, and neuromyelitis optica spectrum disease groups were correlated with EDSS score and MRI gadolinium enhancement. The results manifest that the NfL level in the cerebrospinal fluid is conducive to assessing the severity and possible progression of demyelinating diseases (18). In MS patients, there is a correlation between the plasma NfL level and EDSS score, and the EDSS score is strongly correlated with multiple MRI volume parameters (19). Pauwels et al. conducted a prospective cohort study of 115 MS patients and 30 controls, and disease deterioration was defined as an increase in at least one of three measures (EDSS score, timed 25-foot walk, and 9-hole peg test). They found a significant correlation between the plasma NfL level and disability deterioration (20). Scholars from outside of China have detected the NfL levels of MS patients and conducted long-term follow-ups. Patients with high baseline NfL levels have a significantly high risk of developing EDSS.

The results in this study illustrate a positive correlation between the EDSS score and the plasma NfL level, meaning that the higher the EDSS score, the higher the plasma NfL level, possibly due to two aspects. First, there is persistent chronic inflammation in the CNS of patients with MS. Although active lesions or disability progression was not found in most patients through MRI scans and EDSS scores, this chronic injury can cause continuous axonal damage and release NfL into the cerebrospinal fluid and blood. Second, in the later stage of the disease, the compensatory repair effect of patients diminishes, and the axonal regeneration ability weakens. Therefore, the NfL content in the cerebrospinal fluid and blood of MS is significantly high. It suggests that NfL can be used to some extent for assessing MS severity and is an essential indicator of disability progression in patients.

The diagnosis of MS recurrence primarily relies on MRI gadolinium enhancement and clinical recurrence. Some patients do not have typical clinical manifestations of recurrence, and some patients only present discomfort or limb numbness, making it difficult to determine whether there is a recurrence. Moreover, even false seizures exist in some patients. Enhanced MRI is an objective indicator. However, there still may be omissions due to limitations. The role of NfL in monitoring disease activity and severity is extensively recognized. Some reports have shown a strong correlation between the NfL level and the number of active lesions on MRI. The disease activity caused by active inflammation (new T2 and gadolinium-enhanced lesions) is a critical factor in the elevation of NfL. A prospective cohort study included 58 Canadian and Italian MS patients. In this study, patients with active MS were followed up every 3 months or less for 1 year, including clinical evaluation, MRI scanning, and serum extraction. Quantitative analysis was performed using the Simoa platform. NfL with a high baseline was associated with future recurrence, MRI lesions, compound recurrence-related deterioration, and progression independent of recurrent activity (21). After the acute recurrence of MS, NfL is damaged and released into the cerebrospinal fluid and peripheral blood, consistent with MRI recurrence. In a 2018 prospective study involving 259 patients with MS, it was found that for each enhancement lesion in MS patients, the NfL level increased by 17.8%, and for each new or enlarged T2 high signal lesion, the sNfL level increased by 4.9%. However, it was not correlated with the volume of T2 lesions (22). In this study, the number of MRI-enhanced lesions in patients in the acute stage was higher than that in patients in the remission stage, representing that enhanced lesions are essential for seizure determination. The number of MRI-enhanced lesions was positively correlated with the plasma NfL level. The more MRI-enhanced lesions, the higher the plasma NfL level, indicating that enhanced lesions reflect the activity of inflammation and are related to the severity of axonal injuries. Therefore, an increase in the NfL level may imply the recurrence of the disease.

Recent studies have proven that an increase in NfL during the clinical stage of MS may be an ideal prognostic biomarker for predicting disease progression and guiding treatment decisions. As visual evoked potential (VEP) is increasingly used as a quantitative parameter for myelin sheath in clinical trials, scholars evaluate the correlation between VEP latency and retinal neurodegeneration and prognostic potential in RRMS using optical coherence tomography (OCT). P100 latency can indicate disease prognosis and is correlated with NfL at the baseline (23). NfL levels increase 6 years before the clinical onset of MS, representing that MS may have a prodromal stage that lasts for several years, during which axonal damage has already occurred. Therefore, the main pathogenesis of MS may not be inflammation but neurodegeneration. It can lead to irreversible neurological deficits. This transformation is a key factor in long-term prognosis (24). With the approval of efficient disease modifying therapies (DMTs), some of these drugs have verified effects on neurodegeneration. Therefore, there is an urgent need for reliable biomarkers to identify this transitional stage early and actively intervene in high-risk progressive patients.

MRI is irreplaceable in diagnosing MS, monitoring clinical medication, and evaluating suspected recurrence or disability deterioration (25). MRI is the gold standard for diagnosing patients with MS and the most powerful clinical tool (26). The 2021 European Neurology Annual Conference brought many advancements in iconography. The conference expounded that in patients with MS, massive T2 lesions of large volume, the occurrence of gadolinium-enhanced lesions and spinal cord lesions, whole brain atrophy, and gray matter atrophy were observed, all of which predicted poor prognosis (26). To analyze which lesion sites on MRI were most closely associated with the cerebrospinal fluid NfL level, Adams et al. performed a lumbar puncture, collected the cerebrospinal fluid from 139 patients with MS, and followed 25 of them. The cerebrospinal fluid NfL and MRI relationship was evaluated based on the location and number of lesions. Spearman rank correlation was used to assess the correlations between the cerebrospinal fluid NfL and MRI lesion location, as well as baseline and 1-year follow-up lesion count. The results suggest that the correlations between the baseline cerebrospinal fluid NfL and lesion location and subsequent lesion are general, while the correlation between baseline MRI and the cerebrospinal fluid NfL is strong: periventricular, juxtacortical, infratentorial, and spinal cord injuries (27). It can be seen that there is a weak positive correlation between the cerebrospinal fluid NfL and typical periventricular lesions of MS, a moderate positive correlation between juxtacortical lesions and the cerebrospinal fluid NfL, and a strong positive correlation between the cerebrospinal fluid NfL and infratentorial and spinal cord lesions. This study found in clinical work that MS patients with MRI infratentorial and spinal cord lesions usually had poor prognoses. Therefore, the MRI examination of the head, cervical, and thoracic spinal cord was performed on patients with MS, and the number of T2 lesions was counted. According to rank correlation analysis, there is a correlation between the plasma NfL level and the number of MRI T2 infratentorial and spinal cord lesions. In the 2020 EMotion Forum, experts affirmed the role of plasma NfL in diagnosing MS and its potential for evaluating prognosis (28). This study shows a positive correlation between the number of MRI infratentorial and spinal cord lesions and the plasma NfL level, demonstrating that poor prognosis may exist in MS patients with infratentorial and spinal cord injuries. This study suggests that elevated NfL may be one of the indicators for poor prognosis in patients with infratentorial and spinal cord lesions. However, long-term follow-up is required for further verification.

Significant breakthroughs have been achieved in MS treatment in the past 25 years. As more and more DMT drugs enter the Chinese market, the main problems faced by doctors are deciding who should receive treatment, for how long treatment should be given, and whether to choose high-efficient or low to medium-efficient DMT drugs. These decisions are usually based on treatment tolerance and reasonable expectations for long-term efficacy (29). The plasma NfL level can indicate prognosis and help, to some extent, clinical doctors make optimal choices.

MS is a rare disease in China. The sample size of this study is relatively small. Only patients with MS from the Inner Mongolia Autonomous Region were selected, possibly leading to selection bias. Increasing the sample size and expanding the selection range is necessary in future studies. The present study only followed some patients for 6 months to 1 year. Due to time constraints, long-term follow-up was not conducted. So far, there has been increasing research on liquid-phase biomarkers of MS. Existing studies have demonstrated NfL’s sensitivity to disease activity and status assessment as a biological counterpart for CNS axonal injury, manifesting its potential applicability in MS. Therefore, in the future, whether NfL can be used as a routine examination for MS in clinical diagnosis and treatment still needs to be verified through large-scale longitudinal cohort studies of different populations, more standardized detection methods, time point, and critical value, and the combination with MRI, thus assisting clinic-customized medical practices of MS.

## Data availability statement

The raw data supporting the conclusions of this article will be made available by the authors, without undue reservation.

## Ethics statement

The studies involving humans were approved by the Inner Mongolia Medical University Affiliated Hospital, no. WZ2023056. The studies were conducted in accordance with the local legislation and institutional requirements. The participants provided their written informed consent to participate in this study.

## Author contributions

FM: Conceptualization, Methodology, Writing – original draft. YW: Data curation, Writing – review & editing. WC: Writing – review & editing. YC: Writing – review & editing. XY: Writing – review & editing.
